# Sperm swimming behaviors are correlated with sperm haploid genetic variability in the Mexican tetra, *Astyanax mexicanus*

**DOI:** 10.1371/journal.pone.0218538

**Published:** 2019-06-26

**Authors:** Richard Borowsky, Alissa Luk, Rebecca S. Kim

**Affiliations:** 1 Department of Biology, New York University, New York, New York, United States of America; 2 Department of Environmental Medicine, Langone Center, New York University, New York, New York, United States of America; University Hospital of Münster, GERMANY

## Abstract

The diploid genotypes of males are widely thought to determine sperm phenotypes, yet recent work shows that the haploid genetics of the individual sperm cell also contributes significantly. We tested seven sperm phenotypes, flagellar length and six behaviors, looking for correlations between genetic and phenotypic variability. While flagellar length appears to be controlled by the diploid genotype of the source, variation in three of the behavioral phenotypes, linearity, wobble, and progression are significantly correlated with the heterozygosity of the male producer. Because males that are more genetically variable produce a sperm set that is more diverse in its haploid genotypes, we suggest that the correlations may reflect significant haploid genetic control of sperm swimming behaviors.

## Introduction

Sperm cells from the same ejaculate (sib-sperm, *sensu* Sivinski 1984 [1]) exhibit remarkable diversity in behavior and morphology. Many are immotile, and among those that can swim there are radical differences in swimming speed and trajectory [2]. In species where the sperm normally have a single flagellum, significant proportions have abnormal morphology, including having two heads, or various numbers of flagella, etc. [3]. High proportions of such atypical cells have been documented in sperm from a broad range of animal species, including humans [3–11]. Thus, this variation could be viewed as “normal” for sperm cell populations. The World Health Organization study on sperm characteristics of fertile men found that the median percentage of sperm failing to exhibit progressive motility was 45% and that the median percentage of sperm with abnormal morphologies was 85% [12]. This is important because most abnormal cells appear to be incapable of fertilization [4]. Thus, whatever genes they carry are excluded from the next generation.

Why do ejaculates normally have a high proportion of non-functional sperm cells? One explanation is that it is difficult to manufacture defect-free sperm because the cell type is so complex in form and function. In this view, non-functional sperm are manufacturing defects that have escaped quality control (2) and are thus a maladaptive waste of resources. Alternatively, however, non-functional cells may actually be an important adaptation. If alleles that would be detrimental to a zygote also produce sperm that cannot effect fertilization, then other sperm, with more robust genetics, would be more likely to deliver their alleles to the egg. This would optimize the fitness of the zygote and reduce the number of deleterious alleles in the next generation. [13].

The haploid genotype of each sperm cell is unique, thus allowing ample genetic variation among sib-sperm cells to account for their high phenotypic variation. However, the current view is that the diploid genotype of the male is the major, if not sole, genetic determinant of sperm phenotype. This view comes from the observation that developing spermatids share cytoplasm through intercellular bridges which facilitate homogenization of their gene product contents. Therefore, sperm cells, while “genetically haploid”, are said to be “phenotypically diploid” [14]. It is possible that the effect of the diploid genotype is directly mediated in the primary spermatocytes or indirectly through influences during development from the diploid Sertoli cells.

Recent work, however, shows that the situation is more complex, and a sperm cell’s haploid genotype can influence its phenotypes. Alavioon *et al*. [15] sequenced the genomes of zebrafish sib-sperm subpopulations and documented genome wide allelic (SNP) differences between the pooled genetics of strong *vs*. weak swimmers. In another study, subpopulations of sperm with different responses to a chemical challenge differed in allelic content, and a haplotype already having been shown to have poorer fertilization success was enriched in the more fragile subpopulation [13]. Thus, there is growing evidence that at least some sperm genotypes are correlated with sperm phenotypes. Our working hypothesis is that the diploid genotype of the male and the haploid genotypes of its sperm both affect sperm phenotype. The question is what are the relative contributions of these two sources of genetic information for any particular phenotype? This can be tested.

The haploid control hypothesis predicts that the sperm of more heterozygous males would have greater phenotypic diversity than those of less heterozygous males, because of their greater genetic diversity [16,17]. We used this prediction to test the relative effects of diploid and haploid control on several sperm phenotypes. We studied a vertebrate fish model, *Astyanax mexicanus*, the Mexican tetra, measuring flagellar length and sperm swimming phenotypes, representing both morphological and behavioral phenotypes.

In the wild, *A*. *mexicanus* consists of numerous independent populations which vary considerably in heterozygosity. This natural variation facilitates the investigation of potential correlations between male heterozygosity and the phenotypic variation of their sperm. Of further interest, overall phenotypic variation among populations in this species has a strong adaptive component, because some populations reside in open waters while others inhabit caves and have undergone extensive genomic alteration in their adaptation to cave life [18]. Thus, we could ask whether cave populations, as a group, differ from the surface population in sperm characteristics. Furthermore, the various known populations of this species fall into two distinct lineages (“new” and “old,” based on when they were present in the surface streams of the area [19,20]). The lineages are sister taxa and both contain cave and surface populations. Using this system, we tested whether specific sperm phenotypes were lineage-specific.

## Materials and methods

### Expected heterozygosities (H_e_) for non-hybrid and hybrid males

The non-hybrid males used in this study were wild-caught or F_1_ of wild-caught individuals. Thus there was no inbreeding in the group of test males and the heterozygosities of their populations of origin (H_e_) are unbiased estimates of their genetic heterogeneity. Heterozygosities were determined in a previous study of *A*. *mexicanus* natural populations [19]. Data from F_ST_ and H_e_ values from the same study allowed us to calculate heterozygosity values for the two-population hybrids, using the relationship F_ST_ = (1 –H_s_/H_t_) (Equation 3 in [21]), and solving for H_t_ as the best estimate of H_e_ for a hybrid swarm. We calculated the estimate of H_e_ for the four-cave hybrids ([Tinaja X Molino] X [Pachón X Toro]) in two ways: first, by estimating H_e_ for both of the two-cave hybrids separately and then averaging them. This gave a minimum estimate of 0.791. As a maximum estimate we chose 1.0. We used both values to test for correlation between flagellar lengths and heterozygosities. The two estimates gave nearly identical results. The collections were originally made for another study and the details are documented elsewhere [19].

### Sperm collection

Prior to sperm collection, the temperature in the tank holding the males was raised from 20˚C to 26˚C and the males were maintained at elevated temperature overnight. The rise in temperature stimulates production of new sperm [22]. The fish were anesthetized using 0.025% tricaine-methanesulfonate (MS-222, Western Chemical) for about 60 seconds. The males were placed on their backs on a wet pad of nylon wool and gently squeezed to expel the sperm. Sperm was collected in Hank’s buffer [23] in order to suppress activation and were used either for the measurement of sperm flagellar length or for behavioral study and CASA analyses.

### Sperm flagella measurements

In order to measure the tail lengths of individual sperm cells, a 5μL portion of freshly collected sperm suspension was pipetted onto a glass slide (25 X 75mm), covered with a cover slip (22 X 30mm), and viewed using negative phase contrast at 400x magnification. Sperm were photographed using a 1.3 MP Monochrome camera (CMLN-13S2M-CS, 1296x964 res, FLIR Inc.) and FlyCap2 software (FLIR Inc.). Sperm tail lengths were measured from the images using the segmented line tool In ImageJ software (NIH). Because the head and the tail were not always in the same focal plane, we could not reliably measure the total length in all cases. Instead, we measured from the center of the midpiece to the end of the flagellum. *Astyanax mexicanus* sperm heads are circular and average 3.8 μm in diameter [13]. Thus total lengths, including the head and half of the midpiece length, would be approximately 5μm more than the average values reported in S1 Table.

### Sperm swimming analyses

#### Video observation

Semen was collected as previously described in Hank’s buffer to prevent premature activation of the sperm. To activate the sperm, a 2μL sample was mixed with 18μL of system tank water by pipetting the mixture in and out for several seconds. Within 15 seconds post-activation, a 0.75μL portion of the activated sperm was pipetted into a single well of a 12-well Multi-test Slide (MP Biomedicals, Irvine, CA, USA), covered with a cover slip, and visualized with a negative-phase contrast microscope (Olympus IMT-2) at 200x magnification. Each video was recorded at 100 frames per second (Chameleon3-U3-13Y3M-CS, 1280x860 res) and the record was analyzed for one second intervals at both 20 and 30 seconds post-activation. A Free Video to JPG Converter was used to convert the 100 frame records to stacks of 100 jpeg images for CASA analysis [24].

#### Computer Assisted Sperm Analysis (CASA) analysis

We processed each stack of images (100 frames) through ImageJ first by using the Subtract Background function at 5 pixel setting, and then setting the Threshold parameter so only the sperm heads were selected. Each stack was then analyzed by CASA, with the operating parameters optimized to track all sperm and to exclude debris (Table 1). The CASA plugin automatically tracks the movement of sperm within a stack of frames and generates pathways for each individual. From these pathways, CASA calculates and reports a variety of parameters to describe sperm motility. For each trait analyzed (VCL, VAP, VSL, LIN, WOB, and PROG, see Table 2 for descriptions of traits). We calculated the mean, standard deviation, and the coefficients of variation (CV) independently for each male. We calculated partial correlation coefficients between CV and heterozygosity for each parameter correcting for the effects of lineage. Similarly, we calculated partial correlation coefficients between lineage and swim trait variability, correcting for the effects of heterozygosity. Nominally significant correlations were corrected for multiple comparisons using the Holm-Bonferroni method [25].

**Table 1:** Parameters for CASA analyses.

Parameter	Setting
Minimum sperm size (pixels)	5
Maximum sperm size (pixels)	100
Minimum VSL for motile (μm/s)	0.1
Minimum VAP for motile (μm/s)	0.1
Minimum VCL for motile (μm/s)	0.1
Frame rate (frames per second)	100
Microns per 100 pixels	625
Print motion characteristics…	1

CASA operating parameter settings optimized for tracking Mexican cavefish sperm. Parameters displayed above are those modified from the original default settings for this analysis.

**Table 2:** CASA phenotypes.

Characteristic	Description
Velocity curvilinear (VCL)	The total distance traveled during a time interval
Velocity average path (VAP)	A moving average of calculated velocity (averaged over 16 frames).
Velocity straight line (VSL)	Velocity measured from the first to the last point reached during the time interval
Linearity (LIN)	VSL/VAP, describes sperm path curvature
Wobble (WOB)	VAP/VCL, describes side-to-side movement of the sperm head
Progression (PROG)	The average distance of the sperm from the origin during all frames analyzed.

Description of phenotypes determined from CASA analyses of sperm movement. (Modified from Wilson-Leedy and Ingermann [26]).

#### Statistical analyses

Raw data compilation was done using Excel (Microsoft). Routine calculations, correlation and partial correlation analyses were done using Statistica 6.0 (Statsoft). Regression analyses were done in Excel using the application Xlstat (Addinsoft).

## Results

### Flagellar length

**Fig 1:**
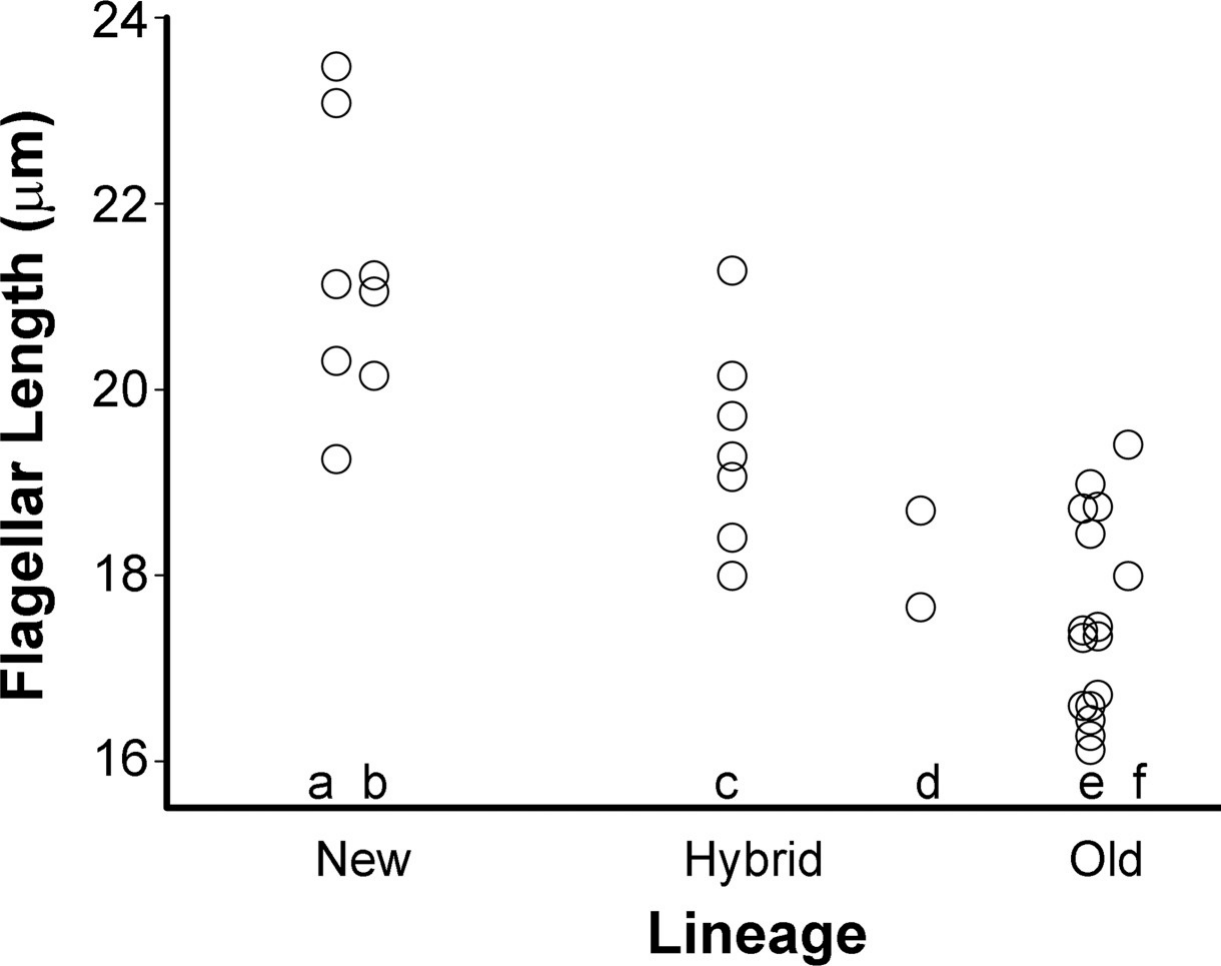
The average length of flagella of sperm from males of two different lineages in *Astyanax* m*exicanus* and their hybrids. Each circle represents the average length of measured sperm from a single male. Sample sizes are given in S1 Table. The New lineage is represented by “a” (cave fish) and “b” (surface fish). Purebred Old lineage is represented by “e” (cave fish). Hybrids between old and new lineage fish (“c”) are intermediate in flagellar length. Hybrids between two old lineage cave populations (“f”) cluster with pure bred Old lineage cave fishes. Hybrids that contain three doses of Old lineage and one dose of New lineage (“d”) are intermediate between Old:New 1:1 hybrids (“c”) and Old lineage fish (“e” and “f”). Individual values in column d were dithered to make them more easily distinguished. Details are in S1 Table.

We measured tail lengths of sperm from 22 males from eight different natural populations ranging in population heterozygosity (H_e_) from 0.4 to 0.83, and 11 hybrid males with calculated heterozygosities ranging from 0.58 to 0.91 (S1 Table; raw data in S2 Table). Values of H_e_ were obtained from a previous study of SNP variation among *A*. *mexicanus* populations in nature [19], or calculated as described in Materials and Methods. Flagellar length for sperm of eight males in the new lineage averaged 21.2μm ± 0.51μm SEM while the sperm of 14 males in the old lineage differed significantly from them with length averaging 17.4μm ± 0.26μm SEM (t_20_ = 7.44, p < 1E-6). Hybrids between the old and new lineages produced sperm with flagella intermediate in length between those of the two lineages, 19.4μm ± 0.42μm SEM, and significantly different from both, (hybrids vs. “new”: t_13_ = 2.69, p = 0.019; hybrids vs. “old”: t_19_ = 4.30, p = 0.00038 (Fig 1).

Two hybrids between cave populations of the same lineage (“old”) had sperm with tail lengths matching those of the pure old lineage (18.7μm ± 0.71μm SEM; Fig 1). Two other hybrids that were genetically three quarters old lineage had sperm tail lengths almost exactly intermediate (18.2μm ± 0.52μm SEM) between those of the 1:1 Old:New hybrids and non-hybrid Old lineage males (Fig 1). Thus it is likely that the key factor explaining variation of flagellar length among populations and hybrids is the allelic content in the diploid males from the two lineages (Fig 1).

We did correlation analyses, coding new lineage males as 1, old lineage males as 2, and hybrids as 1.5 or 1.75, depending upon lineage content. The correlation between lineage and average sperm length for all 33 males was highly significant (r = -0.811, n = 33, t_31_ = 7.71, p = 1.1E-8). Limiting consideration to the pure lineage males, the correlation was similar in magnitude and also highly significant (r = -0.857, n = 22, t_20_ = 7.44, p = 3.5E-7). These results confirm lineage as the major correlate of flagellar length variation in this sample of males.

To determine whether within-male variation in flagellar length is also correlated with genetic heterozygosity, we calculated coefficients of variation (CV) of flagellar lengths for each male. For all 33 males studied, the correlation between CV and H_e_ was -0.060 (p = ns). Calculated separately, by lineage, the correlation coefficient for the eight new lineage males was -0.468 (p = ns), for the 14 old lineage males it was +0.548 (t_12_ = 2.27, p = 0.042), and for the seven 1:1 lineage hybrids it was -0.34 (p = ns). While the value of +0.548 is marginally significant (p = 0.042), its evidence is far too weak to reject the null hypothesis of no correlation, given that the correlations for the other groups are both of opposite polarity (negative), and overall there is no significant correlation. We conclude that the heterozygosity of the male producing the sperm has no significant effect on variation in tail length. Our results are consistent with previous observations and conclusions [17] that sperm lengths are controlled by the diploid genotypes of the males producing them, with no evidence of influence from the haploid genomes of the individual sperm.

### Sperm behavior

We used a Computer Assisted Sperm Analyzer program (CASA, [26,27]) to characterize patterns of sperm behavior to test whether they are controlled in any significant way by the haploid genomes of the individual cells (Materials and Methods). We checked whether variation in sperm phenotype is correlated with the genetic variability of the males producing the sperm. Because more heterozygous males produce more genetically diverse sperm, a positive correlation is expected when a phenotype is determined by the sperm’s haploid genotype.

We examined sperm phenotypes computed by CASA for one second intervals at 20 and 30 seconds post-activation (data in S3 Table and S4 Table). For each of the six phenotypes, we computed the mean values for sperm in each ejaculate, standard deviations, and coefficients of variation (CV).

We did Principal Component Analyses on the six behavioral traits for each time period. The first PCA axes at both times capture the bulk of the variance in the data set (85.0% for the 20 second data and 75.1% for the 30 second data). Thus the PCA vectors are appropriate as overall indicators of phenotypic variability. Multiple regression and partial correlation analyses were performed to test the significance of correlations between these PCAs and heterozygosity, with lineage partialed out, and vice-versa. For the 20 second data, correlations between the PCA1 and both heterozygosity and lineage were significant (Table 3). For the 30 second data the correlation between PCA1 and heterozygosity was significant, but not between PCA1 and lineage.

**Table 3:** Variability of some sperm phenotypes is positively correlated with genetic variability among sperm haploid genotypes.

		PCA1	VCL	VAP	VSL	WOB	LIN	PROG
20 Sec	r =	**0.384**	0.25	0.272	0.293	0.435*	0.413*	0.258
H_e_	t =	**2.66**	1.65	1.81	1.96	**3.09**	**2.9**	1.71
	p =	**0.011**	0.106	0.079	0.056	**0.0036**	**0.006**	0.095
20 Sec	r =	**0.407**	0.503*	0.437*	0.403*	0.201	0.221	0.106
Lineage	t =	**2.86**	**3.72**	**3.11**	**2.83**	1.38	1.45	0.68
	p =	**0.0067**	**0.00059**	0.0034	**0.0073**	0.176	0.155	0.5
30 Sec	r =	**0.395**	**0.316**	0.275	0.171	0.399*	**0.328**	0.417*
H_e_	t =	**2.72**	**2.1**	1.8	1.1	**2.75**	**2.19**	**2.9**
	p =	**0.0096**	**0.042**	0.078	0.279	**0.0089**	**0.034**	**0.006**
30 Sec	r =	0.195	**0.335**	0.217	0.179	-0.116	-0.034	0.32
Lineage	t =	1.34	**2.25**	1.41	1.15	-0.74	-0.216	2.14
	p =	0.187	**0.03**	0.167	0.256	0.463	0.83	0.039

Partial correlations between heterozygosity of the male producing the sperm and its lineage and the variability of the sperm swimming phenotype. Sperm behaviors: VCL = curvilinear velocity, VAP = average path velocity, and VSL = straight line velocity. Wobble (WOB) = VAP/VCL and describes side to side movement of the head; LIN = VSL/VAP and describes path curvature; PROG = progressivity. The partial correlations (r) represent the correlations of He, controlled for lineage effects, or vice versa, with the six swimming traits and their first PCA. Nominally significant correlations are shown in bold face.

*denotes those correlations significant after correction for multiple comparisons (Holm-Bonferroni).

**Fig 2:**
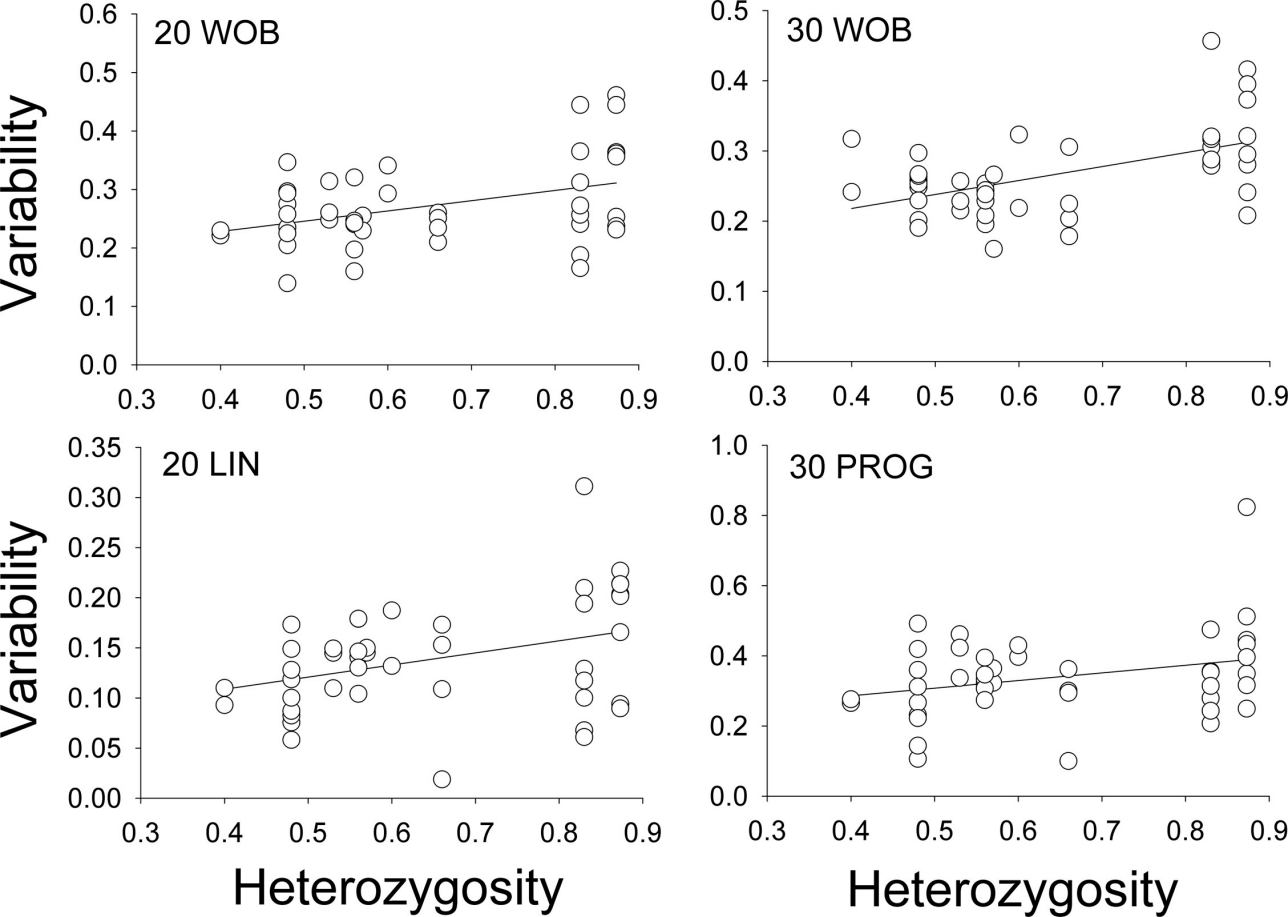
Significant relationships between sperm behavior as quantified by CASA and heterozygosity. Plotted are coefficients of variation (CV) in WOB at 20 and 30 seconds, LIN at 20 seconds and PROG at 30 seconds, against expected heterozygosities of the male sources of the sperm.

Given the significant correlations between overall phenotypic variability and heterozygosity, we looked for correlations between each individual swim phenotype and heterozygosity (corrected for lineage). We controlled for multiple comparisons using the Holm-Bonferroni method [25]. We found that both WOB and LIN were significantly correlated with heterozygosity in the 20 second data as were WOB and PROG in the 30 second data (Table 3; Fig 2). We suggest that these significant correlations reflect at least partial haploid control of the three sperm behaviors.

Turning our attention to the correlations between lineage and phenotype in the 20 second data, we found significant (post-Bonferroni) correlations with three swim traits: VCL, VAP, and VSL. Of interest is that none of these three traits correlates, even weakly, with heterozygosity. We return to this point in discussion.

The data vectors, the principal component analyses and the regression analyses are shown in S5 and S6 Tables. The raw data from CASA are available in S3 and S4 Tables.

## Discussion and conclusions

Sperm phenotypes are currently believed to be determined primarily, or solely, by a male’s diploid genotype. The developing spermatids are joined by cytoplasmic bridges through spermiogenesis, and it is argued that any haploid genetic differences are swamped during this syncytial period [14]. Recent work, however, shows that sperm phenotypes may be significantly determined by sperm haploid genotypes [15,28]. To investigate the potential influence of a sperm’s genotype on its phenotype, we studied the flagellar length and behavior of sperm from *A*. *mexicanus* males of different lineages and different degrees of heterozygosity.

If sperm phenotypes are even partially determined by their haploid genotypes we would expect that sib-sperm from males with high heterozygosity would be more phenotypically variable than those of males with low heterozygosity [17]. We tested this prediction with a morphological phenotype, flagellar length, and six different swimming traits.

For flagellar length we found no evidence supporting this prediction. Male heterozygosity did not correlate with variability in tail length of sib-sperm. Instead, we found that tail lengths differed between two distinct lineages and were intermediate in hybrids between them. Thus, we conclude that tail length in *A*. *mexicanus* is determined by the diploid genetics of the male producer and not by the haploid genetics of the individual sperm cells. This is in accord with previous observations on fly sperm [17].

Our conclusions for sperm swim behavior are very different, however. We analyzed the swimming behavior of the sperm from 44 males of varying heterozygosity values, from 0.4 to 0.873; the greater a male’s heterozygosity, the more genetically diverse its haploid sperm genotypes. We used CASA to quantify six swimming phenotypes of the sperm. Three of the phenotypes, wobble (WOB), linearity (LIN), and progression (PROG), all plausibly related to fertilization success, exhibited significant correlations between trait variability within an ejaculate and heterozygosity of the male producer. The most parsimonious explanation for these correlations is that these swim characteristic are significantly determined by the haploid genetics of the individual sperm.

We also found significant correlations between lineage (corrected for heterozygosity) and variation in three swim phenotypes, VAP, VSL and VAP. Variations in these traits are not correlated with heterozygosity. Thus, it is likely that these correlations stem from diploid differences between the lineages, much as we concluded for tail length. These correlations were evident in the 20 second data but not at 30 seconds (Table 3). These correlations were unexpected and warrant further study.

Tail length and swim phenotypes are expressed at different stages of sperm development. The development of the tail, and its final length, is completed while the four spermatids of the tetrad are still attached by cytoplasmic bridges. Likely, this interconnectivity minimizes the differences among the haploid genotypes of the individual sperm so that the only detectable genetic influence is the diploid genotype of the male producer. However, swimming behavior occurs after the sperm are separated from each other and are fully mature. This might permit a sperm’s unique haploid genotype to be expressed and to affect swimming behavior.

A potential alternative explanation for the correlation between male heterozygosity and variability of swimming traits is that more heterozygous males might simply produce more variable sperm. One mechanism for this could be stochastic gene expression and incomplete penetrance, as has been documented in cell fate variability in the retina [29,30]. Such a mechanism could produce significant phenotypic variation among Sertoli cells, for example, in spite of their identical diploid genetics. Such differences could have phenotypic consequences for the sperm. Arguing against this interpretation, our data show no correlation between genetic variability and variability in flagellar length although, of the seven phenotypes we studied, flagellar length, determined while the spermatids are still syncytial, is the one most likely to be shaped by the diploid genotype of the male. The same result was obtained in a previous study of flagellar length and its variability in two species of flies. The study contrasted the variability of lines selected for short or long flagella and their hybrids and found no correlation between male heterozygosity and trait variability [17]. Thus, in a trait very likely to reflect diploid control there is no empirical evidence that more heterozygous males produce more variable sperm.

For its haploid genotype to affect development and behavior of a sperm cell there would have to be a mechanism for its information to be read out, presumably through transcription and translation. Sperm contain a rich mixture of mRNAs, many of which are post-meiotic in origin [31–35]. Because transcription of thousands of genes proceeds into the spermatid phase in animals it is possible that the transcription profiles of individual cells could diverge based on their haploid genotypes.

## Supporting information

S1 Table The average lengths of flagella in phylogenetically distinct lineages of *Astyanax mexicanus*. Lineages are defined in Borowsky and Cohen, 2013 [18]. Each row lists the statistics of the sperm from a single male. The number of sperm measured per male = n. Average lengths, standard deviations of length, coefficients of variation (CV) and population heterozygosities (H_e_) (see Methods) are in the last four columns. Cave population locations are listed in Mitchell et al. 1977 [36]. Some cave names are abbreviated in the table; full names are: Molino, Caballo Moro (CMoro), Yerbaniz (Yerb), Tinaja (Tina), Pachón (Pach), Arroyo, Toro, Chica, and Curva. The “4 Hybrid” males were hybrids of two hybrid crosses: [Tinaja X Molino] X [Pachón X Toro].
(DOCX)

S2 Table Raw data and analyses of variation in sperm tail lengths from males of different cave and surface populations of *Astyanax mexicanus*. (XLSX)

S3 Table CASA raw data output and analyses of sperm behavior from males from different populations of cave and surface *Astyanax mexicanus* 20 seconds post-activation. (XLSX)

S4 Table CASA raw data output and analyses of sperm behavior from males from different populations of cave and surface *Astyanax mexicanus* 30 seconds post-activation. (XLSX)

S5 Table CASA data and its statistical analyses by principal components and multiple linear regression for the 20 second data. (XLSM)

S6 Table CASA data and its statistical analyses by principal components and multiple linear regression for the 30 second data. (XLSM)
