# Supplementary material for: Sperm swimming behaviors are correlated with sperm haploid genetic variability in the Mexican tetra, Astyanax mexicanus
Source: PLoS One. 2019 Jun 26;14(6):e0218538. doi: 10.1371/journal.pone.0218538 (PMC6594619; doi:10.1371/journal.pone.0218538)
Supplement: S1 Table — Lineages are defined in Borowsky and Cohen, 2013 [18]. Each row lists the statistics of the sperm from a single male. The number of sperm measured per male = n. Average lengths, standard deviations of length, coefficients of variation (CV) and population heterozygosities (He) (see Methods) are in the last four columns. Cave population locations are listed in Mitchell et al. 1977 [36]. Some cave names are abbreviated in the table; full names are: Molino, Caballo Moro (CMoro), Yerbaniz (Yerb), Tinaja (Tina), Pachón (Pach), Arroyo, Toro, Chica, and Curva. The “4 Hybrid” males were hybrids of two hybrid crosses: [Tinaja X Molino] X [Pachón X Toro]. (DOCX) [file pone.0218538.s001.docx]

S1 Table. The average lengths of flagella in phylogenetically distinct lineages of *Astyanax mexicanus*.

Lineage Population n = Avg (μm) Stdev(μm) CV H_e_

New Surface 240 20.15 1.89 9.38 0.83

Surface 296 21.05 1.77 8.42 0.83

Surface 92 21.23 2.31 10.86 0.83

Molino 32 20.30 1.94 9.54 0.4

Molino 53 23.47 3.97 16.90 0.4

Molino 159 21.13 2.41 11.42 0.4

Molino 168 23.08 2.35 10.20 0.4

CMoro 27 19.25 2.27 11.80 0.58

Old/New Yerb/Surface 105 19.05 2.26 11.86 0.861

Hybrids Tina/Moli 112 21.28 2.35 11.04 0.906

Pach/Moli 116 20.14 2.25 11.19 0.846

Pach/Epi 160 18.40 2.28 12.38 0.873

Pach/Epi 129 17.99 1.90 10.59 0.873

Pach/Epi 79 19.71 2.24 11.36 0.873

Pach/Epi 101 19.28 2.15 11.16 0.873

Old Arroyo 77 18.71 2.19 11.70 0.53

Arroyo 15 17.32 1.77 10.22 0.53

Arroyo 48 16.44 2.04 12.43 0.53

Pachón 109 17.34 2.18 12.57 0.48

Pachón 151 18.44 2.39 12.94 0.48

Pachón 131 18.73 2.24 11.98 0.48

Pachón 224 17.40 1.98 11.40 0.48

Pachón 162 16.12 1.81 11.24 0.48

Pachón 131 17.44 1.77 10.17 0.48

Tinaja 112 16.59 1.87 11.25 0.56

Tinaja 120 16.59 1.84 11.11 0.56

Tinaja 75 16.71 2.05 12.24 0.56

Toro 106 18.98 3.33 17.57 0.6

Chica 112 16.27 2.32 14.28 0.64

Old/Old Tina/Curva 104 19.40 2.82 14.53 0.582

Hybrids Tina/Curva 105 17.99 2.47 13.76 0.582

3Old/1New 4 Hybrid 75 18.69 4.23 22.63 0.791 or 1.0

Hybrids 4 Hybrid 267 17.65 1.90 10.74 0.791 or 1.0
